# Do we need “more research” or better implementation through knowledge brokering?

**DOI:** 10.1007/s11625-015-0314-8

**Published:** 2015-06-10

**Authors:** Janet G. Hering

**Affiliations:** 1 Eawag, Swiss Federal Institute of Aquatic Science and Technology, Überlandstrasse 133, 8600 Dübendorf, Switzerland; 2 IBP, Swiss Federal Institute of Technology (ETH) Zürich, 8092 Zurich, Switzerland; 3 ENAC, Swiss Federal Institute of Technology Lausanne (EPFL), 1015 Lausanne, Switzerland

**Keywords:** Knowledge brokering, Knowledge exchange, Research implementation, Science-policy interface, SPI

## Abstract

“More research is needed” is an iconic catchphrase used by scientists worldwide. Yet policy and management decisions are continually being made with variable levels of reliance on scientific knowledge. Funding agencies have provided incentives for knowledge exchange at the interfaces between science and policy or practice, yet it remains the exception rather than the rule within academic institutions. An important step forward would be the establishment and professionalization of knowledge brokering (i.e., as a complement to existing technology transfer and communications departments). This would require an explicit commitment of resources by both funding agencies and institutions. Many academic scientists are genuinely interested in the applications of their research. This interest could be stimulated by providing support for the process of knowledge brokering and by integrating the natural, social, and engineering sciences to address broad policy- and practice-relevant questions.

## Introduction

Continuing concern is expressed in government administrations and funding agencies that policy-making and management do not benefit sufficiently from the knowledge generated by publically funded science (Chapman et al. 2010b; EC 2013; Holmes and Scott 2010; McNie 2007; Pahl-Wostl et al. 2011; Van Enst et al. 2014). This is particularly important in the environmental field, in which most policies and management relate to public goods (e.g., natural resources). Since externalities and market failures are commonplace in cases of public goods, key roles for government and regulation are recognized. This has led to substantial public funding for science that could (or should) contribute to policy-making and resource management decisions although science is, of course, only one of many inputs into decision-making (Choi et al. 2005; Cullen 1990). An estimated 450 water-related projects have been supported by European funding, yet it was noted in the 2012 Roadmap for Uptake of EU Water Research in Policy and Industry (http://www.hydroscan.be/uploads/b117.pdf) that “Unfortunately, the dissemination and uptake of the results of these projects is limited”. Recognizing the need to improve the uptake of research into regulation (specifically the Water Framework Directive), the water directors of the EU and associated States funded an ad hoc science-policy interface (SPI) activity with the goals of identifying relevant available research as well as research gaps and improving the transfer and usability of research (EC 2013).

These concerns and issues are not new and many of the possible remedies identified echo past recommendations (Cullen 1990). In the interest of formulating a path toward effective knowledge exchange at the interfaces of science with policy and/or practice (referred to herein collectively as SP^2^I), it is worthwhile to review briefly the key impediments and measures that have been previously been identified. Although this paper focuses mainly on the academic perspective, cooperation with non-academic partners is essential to effective knowledge exchange.

## Key impediments to effective knowledge exchange are well known

Three key impediments to effective knowledge exchange relate to the accessibility, relevance, and timeliness of research. In the first case, research outputs, which generally appear in the peer-reviewed scientific literature, are not written in a way that is accessible to managers and/or policy makers. In the second case, research fails to provide usable information that is needed for policy and/or management decisions. And, in the third case, even relevant and accessible research outputs may not be available at the time when they would be needed as input to decision-making for policy and/or management (Choi et al. 2005; Kirchhoff et al. 2013; Martini et al. 2013; Opwanya et al. 2013; Sarewitz and Pielke 2007). Cultural mismatches between scientists and decision-makers have also been identified, in particular, that scientists seek to draw recommendations from the weight of the evidence while policy makers often seek evidence to support favored policy solutions (Cullen 1990). These are exacerbated by the lack of personal contact between members of these groups (Choi et al. 2005) as well as by the persistence of linear models of knowledge transfer (Calow 2014; Slob et al. 2007), the disconnect with academic incentive systems (Hering et al. 2012; McNie 2007), and time conflicts with other professional obligations (Pennell et al. 2013).

## Mismatch with the interests of (most) scientists and institutional incentives

These key impediments are easily understandable when they are considered in the context in which academic research is conducted. Publications in the peer-reviewed scientific literature are the “currency” of academia. Despite the recent push-back against the tyranny of journal impact factors (Bladek 2014), academic institutions have made scant progress in defining and applying alternative metrics for promotion and tenure. The acquisition of funding for research is also often strongly tied to the applicant’s publication record. The identification of research topics and the initiation of research projects are, in the ideal, driven by curiosity (Zewail 2010) though the role of opportunity (e.g., through application of new technology) and pragmatic considerations of funding and career advancement cannot be ignored. Critically for the application of research, science and scientists are fundamentally oriented toward questions and new knowledge (Firestein 2012), which implies that meaningful consideration of relevance is likely to receive insufficient attention in the setting of research agendas in the absence of external incentives. Furthermore, the cutting edge of research (characterized by active debate among researchers and, often, of most interest to them) does not usually provide the most useful and usable information for policy and management (Hering et al. 2014; Holmes and Scott 2010).

## Knowledge brokering and boundary organizations as avenues for effective knowledge exchange

**Fig. 1:**
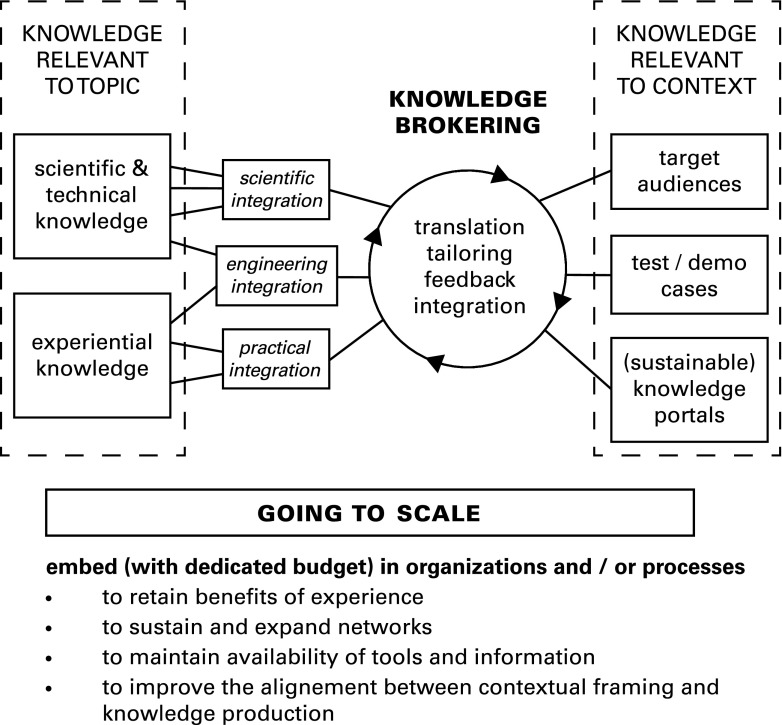
Schematic representation of knowledge brokering positioned as an iterative process of translation, tailoring, feedback and integration that allows information to be exchanged (in both directions) between scientific and technical experts and policy- and decision makers

First and foremost among the various measures recommended for effective knowledge exchange at the SP^2^I is knowledge brokering (also called translation) either within academic research institutions or in separate boundary organizations (Bielak et al. 2008; Cash et al. 2003; Chapman et al. 2010a; Cullen 1990; Kiparsky et al. 2012; Lemos et al. 2012; Martini et al. 2013; McNie 2007; Pennell et al. 2013; Phipps and Morton 2013; PSI-connect 2012; Shaxson et al. 2012; Turnhout et al. 2013; Ward et al. 2009). As outlined schematically in Fig. 1, knowledge brokering is an iterative and bidirectional process of translation, tailoring of information for specific contexts, feedback, and integration. In addition to facilitating the uptake of research into policy and practice, knowledge brokering should help to identify the information that could be useful to support policy decisions so that research can be directed toward filling critical knowledge gaps. To promote information flow in both directions, knowledge brokers must have sufficient relevant expertise to engage with both scientific experts and policy makers or managers and must have a wide range of professional skills, most notably in communication (Phipps and Morton 2013). Knowledge brokers can sustain long-term partnerships with decision-makers that are needed to establish trust (Kirchhoff 2013; Pennell et al. 2013). Although knowledge brokers can be highly effective working within academic institutions, this is often embedded within specific projects without a clear professional outlook after the project ends (Kirchhoff 2013). Scientific experts within academia can act as knowledge brokers; this can be effective within targeted programs such as Cooperative Extension in the US Land Grant Colleges (Osmond et al. 2010) but can also be problematic due to the mismatch with expectations and incentives in academia as well as competing demands on time (McNie 2007; Pennell et al. 2013; Turnhout et al. 2013). Different models for knowledge brokering have been defined (specifically knowledge management, linkage/exchange, and capacity building); these are often combined in practice (Turnhout et al. 2013; Ward et al. 2009). A wide range of tools and concepts for knowledge brokering have been developed and are available through various (and variably maintained) websites (Table 1). Two of these websites, the Knowledge Brokers’ Forum (KBF) and research to action (R2A), provide entry points to on-line communities engaged in knowledge brokering.

**Table 1:** Examples of web resources for knowledge brokering

Name	Type/owner	Focus	URL
Integration and implementation sciences (I2S)	Website managed by ANU faculty	I2S provides concepts and methods for conducting research on complex, real-world problems. It enhances: synthesis of disciplinary and stakeholder knowledge; understanding and management of diverse unknowns; provision of integrated research support for policy and practice change	http://i2s.anu.edu.au/
KStar (K*) initiative	Project website UNU-INWEH	K* is the collective term for the set of functions and processes at the various interfaces between knowledge, practice, and policy. K* improves the ways in which knowledge is shared and applied; improving processes already in place to bring about more effective and sustainable change	http://inweh.unu.edu/kstar/ ^a^
Knowledge Brokers’ Forum	Website managed by the I-K-mediary network	The Knowledge Brokers’ Forum (KBF) is a collaborative space to promote knowledge sharing and dissemination around intermediary work in international development	http://www.knowledgebrokersforum.org/
PSI connect	Project website FP7 EU contract number 226915	Connecting policy and science through innovative knowledge brokering in the field of water management and climate change	http://www.psiconnect.eu/ ^a^
Research to Action (R2A)	Website managed by CommsConsult	An initiative catering for the strategic and practical needs of people trying to improve the way development research is communicated and utilized	http://www.researchtoaction.org/
SPIRAL: Interfacing Biodiversity and Policy	Project website FP7 EU contract number 244035	Goals: improved understanding of why and when more effective science-policy interfaces are needed and allow for identification of some criteria for designing them; identification of good practice and additional actions needed to improve the effectiveness of science-policy interfaces for biodiversity	http://www.spiral-project.eu/ ^a^

^a^Website no longer actively maintained

Knowledge brokering is well established within boundary organizations that operate at the interface between the scientific enterprise and politics or administration (Guston 2001; Osmond et al. 2010). Boundary organizations are hugely diverse, varying in size, scope, source and stability of funding, and legal basis or charter. They range from highly prestigious organizations with long histories such as the US National Research Council (which has an explicit mandate to “improve government decision making and public policy”, http://www.nationalacademies.org/nrc/) to large projects (see Table 1) that can function temporarily as boundary organizations but suffer from a lack of continuity. Boundary organizations can also be nested. For example, UN Water (http://www.unwater.org/) is an inter-agency coordination mechanism established by the United Nations in 2003; it counted 31 members and 34 partners at the end of 2013 (UN Water 2014). Some boundary organizations maintain a neutral and independent position while others either take on an explicit advocacy role or are perceived as advocates, often based on the source of their funding. Many industries, for example, support boundary organizations (i.e., professional or trade associations) to share information among members and sometimes to set common standards but often for outreach and lobbying. Consulting firms can also fulfill many functions of boundary organizations, though their orientation toward client satisfaction and financial constraints can limit their objectivity.

An important role played by some boundary organizations is the establishment and maintenance of knowledge portals. Interactive web platforms or tool-kits can provide a valuable mechanism for the dissemination of information and the creation of on-line communities (Lemos et al. 2012). The accessibility and usability of such platforms may, however, be insufficient for the non-expert user (De Lange et al. 2010); “intelligent filtering” is needed to ensure accessibility and usability (Brunner 2014). The temporary nature of knowledge portals and lack of updating is a significant problem for portals that are developed under the auspices of projects (Opwanya et al. 2013). Clearly, the value of such investments is not optimally captured if products languish on the web in an inactive form or disappear altogether (Blind et al. 2010; Kramer and Schneider 2010).

## The way forward for academic institutions

Research and academic institutions are increasingly called upon to bring their expertise to bear on problems of relevance to policy and practice. These problems often involve complex socio-environmental-technical systems and hence require the solution-oriented integration of the natural, social, and engineering sciences. Knowledge exchange at the SP^2^I is an essential component of increasing the relevance of academic research, which requires the targeted commitment of human and financial resources by research and academic institutions, funding agencies, and government administrations. SP^2^I activities can be explicitly incorporated into relevant projects with a clearly defined scope and dedicated budget (Pennell et al. 2013; Slob et al. 2007). Targeted support will be required to allow early involvement of policy makers, managers, and stakeholders from industry and other interest groups in project planning. This would promote a need-oriented focus for research as well as a balancing of competing interests that could help to avoid later conflicts. Continuity is particularly important since it would allow scientific and technical knowledge to be quickly marshalled in response to events that create “windows of opportunity”. These activities can benefit from previously-developed tools and concepts (PSI-connect 2012; Young et al. 2013) as well as the experience gained from past projects (Martini et al. 2013; Shaxson et al. 2012) if sufficient investments of time and resources are made (Ward et al. 2009). The skills and role of knowledge brokers must be respected and supported; if such individuals are to be embedded in research and academic institutions, their positioning needs to be incentivized, clarified, and incorporated into institutional structures (Phipps and Morton 2013; Shaxson et al. 2012; Turnhout et al. 2013).

Nearly all academic and research institutions house support departments for communications and technology transfer; an analogous department for knowledge exchange could provide an institutional home for knowledge brokers and a platform for the uptake and application of SPI tools and/or support and maintenance of SPI web portals. It would be important that such knowledge exchange departments not operate in the “supply-driven” mode that is characteristic of most institutional communications departments but rather fulfill an active and iterative brokering function. This could promote the identification and prioritization of research needed to support policy development and implementation. Academic and research institutions could also formalize agreements with external boundary organizations to ensure stability and continuity for knowledge exchange activities. In either case, cooperation among boundary organizations and knowledge brokers to share effective concepts, strategies, and practices should be actively promoted. Such harvesting of experience should also include real-world examples of both success and failure (Brunner 2014). Ideally, SP^2^I activities would no longer be ad hoc but rather sustainable and systematic (EC 2013).

Even if knowledge brokering becomes professionalized and established within academic or research institutions, knowledge brokers will need scientific experts as partners. There is dubious value to attempting to force this cooperation. Even within the context of the EU Framework Programs, which have strict requirements for knowledge exchange, surveys have indicated that project participants complied with these requirements reluctantly and often did not follow through (Holmes and Scott 2010). At the same time, there are some scientists who are genuinely interested in the application of their research; they should be supported and encouraged within their institutions (Hering et al. 2012). It is not necessary, and perhaps not even desirable, for scientific experts to take the full responsibility for knowledge brokering (Pennell et al. 2013), but there is a wide variety of ways in which scientific experts can contribute fruitfully to knowledge exchange (Hering et al. 2014; Spruijt et al. 2014). Appropriate support for these interested individuals (i.e., provided by knowledge brokers with relevant information about effective tools and processes) can help them to avoid wasting their time in rediscovering what does and does not work at the SP^2^I. In this context, the engagement of social scientists could provide a better conceptual basis for effective knowledge exchange, providing insight into the processes of policy implementation and political decision-making. It would also be productive for experts from the natural and social sciences to interact with their colleagues in engineering, who have professional experience collaborating with practitioners and stakeholders. Scientists and engineers exhibit different “habits of mind”, partly from natural inclination and partly developed through their training. The engineering habits of mind—systems thinking, creativity, optimism, collaboration, communication, and ethical considerations (Katehi et al. 2009)—are badly needed at the SP^2^I.

Knowledge brokers based in academic or research institutions will also need to establish strong and stable relationships with their counterparts in politics, administration, industry, and other target groups. Cooperation with non-academic boundary organizations will be important to complement and extend the contacts of academic knowledge brokers. The distribution of knowledge brokering activities across various types of organizations should promote the effectiveness of knowledge brokering and reflect the contexts in which knowledge is produced and applied.

Much has been written about the potential for science to contribute to decision-making in policy and management. In the environmental sciences, for example, there is an increasing understanding and acceptance of the influence of human activities on our environment at all scales, up to and including the global scale (Steffen et al. 2011). With this comes the realization that there are “not one but many ecological futures” and that “we must actually design and choose our future” (Priscoli 2004). Since environmental issues are inherently embedded in socio-environmental-technical systems and arise in specific contexts, these aspects cannot be ignored either in the decision-making process or in research that seeks to inform this process. The incorporation of scientific knowledge into decision-making for policy and management cannot guarantee that the best decisions are made and neither is scientific input the only, or even the most important, input to decision-making (Choi et al. 2005; Cullen 1990). Nonetheless, decisions that are inconsistent with the underlying biophysical reality are fundamentally flawed and publically funded science should help to avoid such outcomes. “Going to scale” with knowledge brokering offers the best chance for decision-making in policy and management to benefit from scientific knowledge.
